# Preparation of Air Nanobubble-Laden Diesel

**DOI:** 10.3390/nano15171309

**Published:** 2025-08-25

**Authors:** Jiajun Yang, Xiao Xu, Hui Jin, Qiang Yang

**Affiliations:** 1 School of Mechanical and Power Engineering, East China University of Science and Technology, Shanghai 200237, China; y30230710@mail.ecust.edu.cn (J.Y.); qyang@ecust.edu.cn (Q.Y.); 2 State Key Laboratory of Multiphase Flow in Power Engineering (SKLMF), Xi’an Jiaotong University, 28 Xianning West Road, Xi’an 710049, China; jinhui@mail.xjtu.edu.cn

**Keywords:** nanobubble, diesel fuel, pressurization, depressurization, PTA

## Abstract

This research has successfully addressed the technical challenge of generating nanobubbles in diesel fuel, which inherently lacks hydrophilic structures and charged ions, enabling the effective production of high-concentration nanobubble diesel fuel. This breakthrough lays a solid foundation for subsequent research into the combustion performance and combustion mechanism of high-concentration nanobubble fuels. Furthermore, it holds promising potential to advance high-concentration nanobubble fuel as a viable new type of energy source. A specialized device was designed to generate nanobubble-embedded diesel, and particle tracking analysis with n-hexadecane dilution was employed to quantify nanobubble concentration. The results demonstrate that the nanobubble concentration in diesel increases with both circulation time and pressure, reaching up to 5 × 10^8^ ± 3.1 × 10^7^ bubbles/mL under a pressure of 2.5 MPa. Stability tests indicate an initial rapid decay (50% reduction within one week), followed by a slower decline, which stabilizes at 4.5 × 10^7^ ± 3.13 × 10^6^ bubbles/mL after two months. Notably, nanobubble concentration has a minimal impact on the density and viscosity of diesel but slightly decreases its surface tension. This study presents a feasible method for preparing high-concentration nanobubble diesel, which lays a foundation for investigating the combustion mode and mechanism of nanobubble diesel fuel. With the goal of enhancing combustion efficiency and reducing pollutant emissions, this work further paves the way for the application of high-concentration nanobubble diesel as a new energy source in fields including automotive, marine, and aerospace industries.

## 1. Introduction

Integrating nanobubbles into fuel oil represents a highly promising technical approach, as it reduces the reliance of oil-based fuels on water or emulsified fuels. When nanobubbles are introduced into fuel, they can significantly enhance atomization efficiency even under identical atomization conditions—thereby improving the combustion performance and efficiency of internal combustion engines. Additionally, this method helps reduce emissions of CO_2_, NO_x_, and hydrocarbons. Consequently, the production of high-concentration nanobubble fuel can provide key technical support for subsequent thermal experiments and lay the groundwork for exploring its applications in sectors such as maritime, automotive, and aviation. Exploring the stability of nanobubbles also lays the groundwork for the industrial-scale production and application of nanobubble fuel as a replacement for traditional fuels.

Incorporating hydrogen nanobubbles into fuel oil represents a promising approach to enhancing combustion efficiency and reducing emissions, with particular applicability to gasoline and diesel engines. Owing to their distinctive physicochemical properties, hydrogen nanobubbles can improve fuel atomization and evaporation, thereby facilitating a more thorough combustion process [1]. Studies indicate that hydrogen nanobubbles optimize engine performance across varying load conditions. For instance, a study by Oh et al. [2] revealed that gasoline blends with hydrogen nanobubbles—when adjusted to a stoichiometric air–fuel ratio at constant engine speed—enhanced combustion efficiency at 40%, 60%, and 80% engine loads. At 40% load, the hydrogen nanobubble–gasoline blend achieved a power output of 27.00 kW (a 4.0% increase over conventional gasoline’s 25.96 kW) while reducing brake-specific fuel consumption (BSFC) from 291.10 g/kWh (pure gasoline) to 269.48 g/kWh. As load increased, the blended fuel consistently outperformed pure gasoline in BSFC metrics.

Similarly, Brayek’s research [3] demonstrated that using hydroxygen (a hydrogen–oxygen gas mixture) in spark-ignition engines improves combustion efficiency and reduces emissions. Notably, brake torque increased by 3–12% at low speeds (1000–2500 rpm), with the peak torque rpm shifting from 3000 rpm (pure gasoline) to 3500 rpm (hydroxygen nanobubble, HNB). Fuel consumption decreased by an average of 9.7%, with a maximum reduction of 19% (from 158 g/min to 128 g/min at 3500 rpm). HC emissions dropped by 17–32% across all speed ranges (e.g., from 253 ppm to 171 ppm at 3000 rpm), while CO emissions were reduced by up to 42% (at 1500 rpm)—attributed to hydrogen’s carbon-free nature and oxygen’s promotion of oxidation reactions. CO_2_ emissions decreased by 7–9% due to hydrogen diluting the carbon-based fuel proportion. The hydroxygen mixture, generated via electrolysis, contains both macro- and nanoscale bubbles, eliminating the need for additional hydrogen storage systems and streamlining hydrogen utilization.

The underlying mechanism through which nanobubbles influence combustion is likely attributed to “micro-explosion” [4]. Micro-explosion combustion entails the premixing of gas and fuel, followed by injection through a nozzle. Upon injection, fuel droplets containing dissolved gases or composed of lower-boiling-point liquids undergo flash boiling and break up. Under the high-temperature conditions within the combustion chamber, the dissolved gases inside the droplets first nucleate and vaporize, generating bubbles that cause the droplet surfaces to rupture—either breaking them into smaller droplets or inducing intense oscillations. This phenomenon further atomizes the fuel [5], expanding its contact area with air and thereby promoting more complete combustion [6].

During the combustion process, nanobubbles persist in the liquid fuel even after the initial micro-explosion. As demonstrated in the research by Yarom and Marmurn (2015) [7], nanobubbles can function as active sites for boiling and evaporation. In the subsequent second micro-explosion—dominated by water components—nanobubbles act as nucleation sites, accelerating the micro-explosion of water and intensifying the fragmentation of fuel droplets [7]. Following this second micro-explosion, the fuel droplets transform into an aerosol, while dissolved gases are released from the droplets, forming a nearly homogeneous gas mixture composed of these released gases and fuel vapor. This allows the dissolved gases to enhance the combustion of the fuel aerosol. Since this combustion process is no longer constrained by heat and mass transfer rates in the liquid phase, it approaches an ideal combustion state. Current nanobubble mixture preparation technologies primarily utilize water as the continuous phase and hydrophobic gases as the dispersed phase. These methods commonly employ surfactants in water to enhance microbubble stability, capitalizing on impurities and electrical charges at bubble surfaces to stabilize interfaces [8]. Nevertheless, the micro-explosion principle remains applicable to diesel fuel. Consequently, incorporating nanobubbles into diesel can effectively enhance the fuel’s combustion efficiency.

Hydrogen nanobubble (HNB) technology enables the stable dispersion of hydrogen in fuel in the form of nanobubbles, with the goal of leveraging hydrogen’s inherent advantages to enhance combustion efficiency and reduce pollutant emissions. This technology can promote more complete fuel combustion, thanks to hydrogen’s high combustion rate and low ignition energy. Additionally, hydrogen boasts a wide flammable range and rapid flame propagation speed, which can further improve ignition performance and accelerate flame propagation [2,9,10]. HNB technology also exerts an impact on NO_x_ emissions. The introduction of hydrogen nanobubbles lowers combustion temperatures and results in cleaner combustion products, thereby reducing NO_x_ emissions [11,12]. Moreover, it holds potential for reducing particulate matter (PM) emissions: the complete combustion of hydrogen helps mitigate soot formation, and studies indicate that controlling the combustion process can further decrease smoke and dust generation. Furthermore, if the hydrogen used in HNB technology is produced from renewable energy sources, the technology could also contribute to reducing carbon dioxide emissions [13].

Notably, most organic fuels such as diesel are inherently deficient in hydrophilic structures and contain only negligible amounts of charged ions, two critical factors that collectively present substantial challenges for preparing and stabilizing nanobubbles within diesel matrices. Unlike aqueous systems, where polar molecules and abundant ions naturally support nanobubble formation through electrostatic interactions and hydration effects, diesel’s strong hydrophobicity creates a hostile environment for such processes. This inherent lack of hydrophilic sites means there are no favorable anchors for nanobubble interfaces, while the scarcity of charged ions eliminates the electrostatic repulsion that typically prevents nanobubble coalescence in water. As a result, the conventional stabilization mechanisms relied upon in aqueous environments are effectively disrupted, making it difficult to maintain nanobubble integrity in diesel. Thus, this distinctive constraint necessitates the formulation of specialized strategies to surmount diesel’s intrinsic properties and attain stable nanobubble dispersion in fuel oils.

Due to the Ostwald ripening effect, larger bubbles tend to absorb nanobubbles [14], a phenomenon directly observed via in situ transmission electron microscopy by Jong Bo Park [15]. This process leads to a reduction in nanobubble concentration, as larger bubbles grow at the expense of smaller ones. The vaporization and nucleation of dissolved gases serve as the primary energy source for nanobubble generation, where dissolved gases can form either nanobubbles or larger micron/millimeter-scale bubbles. The energy efficiency of nanobubble formation is determined by the pressure-release flow field during this process.

Notably, Xu et al. investigated pressure nozzles [16] and cyclonic separation techniques [17,18], demonstrating that in swirling flow fields, dissolved gases undergo controlled depressurization. Under the centrifugal force of swirling flow, oversized bubbles can be rapidly separated, effectively mitigating the low nanobubble concentration issue caused by Ostwald ripening. This approach highlights the potential of swirling flow fields as a valuable method for enhancing nanobubble diesel preparation, offering a novel strategy to overcome stability challenges in fuel-based nanobubble systems.

This paper presents the design of a device for large-scale preparation of nanobubble-loaded diesel, with nanobubble concentration in diesel quantified via particle tracking analysis. Experiments confirm the stable existence of high-concentration nanobubbles within the air–diesel system. Through systematic optimization of experimental parameters, this study achieves enhanced nanobubble concentrations and characterizes the decay rate of nanobubble concentration over storage time. These findings establish a critical foundation for the practical application of nanobubble-enriched diesel fuel, addressing key challenges in industrial-scale production and storage stability.

## 2. Materials and Methods

### 2.1. Materials

Grade 0 Diesel Fuel from China oil refinery, pressured air, and pressured nitrogen were used from Shanghai Pujiang Special Gas Co., Ltd., Shanghai, China. n-hexadecane was from Shanghai Naicheng Biotechnology Co., Ltd., Shanghai, China.

### 2.2. Experimental Equipment

The experimental system comprises a nanobubble generator, pressure relief device, bubble column, magnetic flap level gauge, gas flow meter, liquid flow meter, diesel circulation pump, pressure gauge, thermometer, air cylinder, oxygen cylinder, and other auxiliary components.

### 2.3. Experimental Procedure

**Figure 1:**
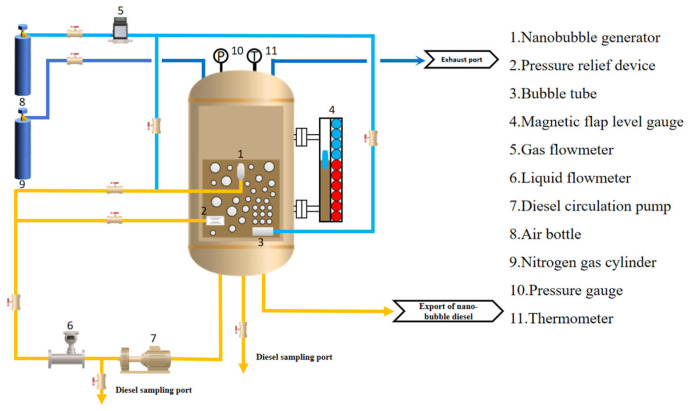
Experimental process diagram.

**Figure 2:**
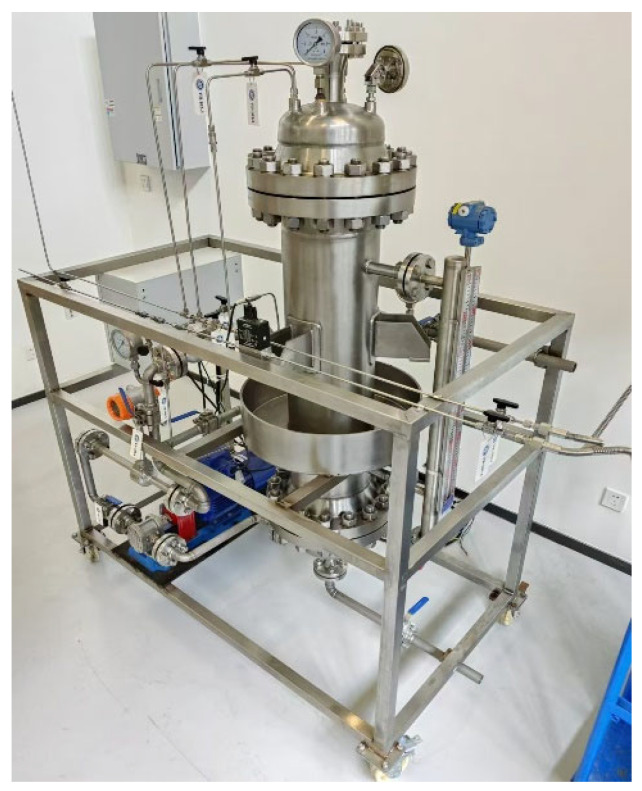
Physical picture of diesel nanobubble generation device.

As shown in Figure 1, this is the experimental process diagram. Guided by this experimental process diagram, we constructed a diesel nanobubble generation device, whose structure is presented in Figure 2. During the experiment, the nitrogen cylinder is first opened to purge the oil storage tank with nitrogen, completely removing the air inside. The gas circuit of the bubbling tube is then activated. The bubbling tube is equipped with numerous 2 mm diameter orifices, through which the gas medium is introduced into the diesel fuel. This dual-function design not only increases the contact area between the gas medium and diesel but also pressurizes the diesel tank via the bubbling tube until the internal pressure reaches the specified working conditions (0 MPa, 2 MPa, and 2.5 MPa).

After setting the gas flowmeter to 50 mL/min and the liquid flowmeter to 15 L/min, the air cylinder and diesel circulation pump are activated. Nanobubble-loaded diesel is prepared through continuous circulation for 1 h. Sampling is performed at circulation time points of 0 min, 4 min, 8 min, 12 min, 20 min, 28 min, 36 min, 44 min, 52 min, and 60 min to measure the nanobubble concentration in diesel at different circulation durations. Curves depicting the relationship between diesel nanobubble concentration and circulation time under varying working pressures (0 MPa, 2 MPa, and 2.5 MPa) are plotted accordingly.

Upon completion of each working-condition experiment, the gas cylinder and diesel circulation pump are turned off, the exhaust valve is opened to release all residual gases, and the experimental procedure is thereby concluded.

### 2.4. Test Method

In accordance with ISO/TR 23015:2020, the particle tracking analysis (PTA) method was adopted for nanobubble measurement, utilizing a Malvern Nano Particle Tracking Analysis Instrument NS300, Malvern, UK. The working principle of the NS300 is as follows: A light source illuminates nanobubbles within the sample, generating scattered light signals that are then captured by the imaging system. The Brownian motion of nanobubbles in the solution is recorded via these scattered light signals. By tracking and analyzing the movement trajectory of the scattered light signal from each individual nanobubble, the particle size and concentration of the nanobubbles are calculated. The NS300 is capable of measuring the concentration of nanobubbles with diameters in the range of 1 nm to 1000 nm. This methodology currently serves as the mainstream technique for quantifying nanobubble concentration in aqueous systems, though its reliability has only been validated in water-based environments to date.

**Figure 3:**
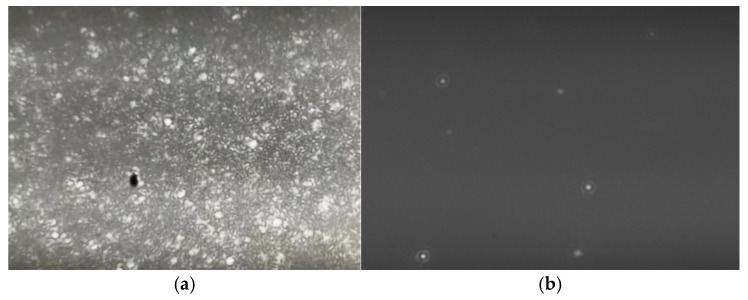
The scattered light spot signal image of nanobubbles captured by NS300 instrument in diesel fuel, (**a**) without dilution step, (**b**) after dilution operation.

**Figure 4:**
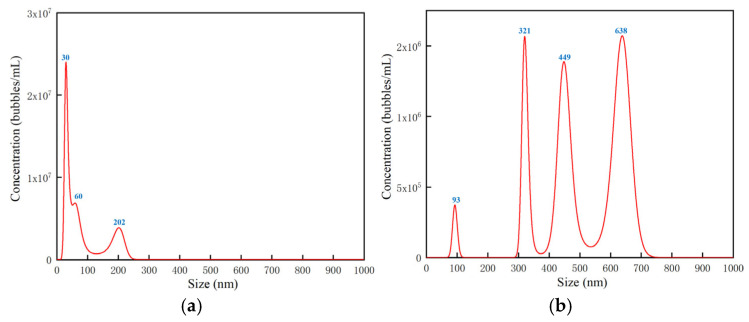
Size-resolved concentration distribution of nanobubbles, (**a**) without dilution step, (**b**) after dilution operation.

Diesel fuel, composed of diverse organic compounds, exhibits a yellowish opaque appearance, as illustrated in Figure 3a. When the NS300 instrument was employed for direct measurement of pure diesel samples, the scattered light spot signal image of nanobubbles captured by the system was filled with numerous snowflake-like noise particles—this severely compromised the visibility of the target signals. The instrument is unable to accurately differentiate between these noise artifacts and diesel nanobubbles, resulting in unreliable nanobubble concentration measurements and poor experimental accuracy and repeatability. Figure 4a presents the size-resolved concentration distribution of nanobubbles, as measured in the diesel medium shown in Figure 3a. The analysis reveals that the NS300 instrument significantly overestimates nanobubble concentration due to interference from abundant noise particles in the diesel background. Furthermore, the detection process exhibits a systematic bias toward smaller nanobubble sizes. As a result, direct experimental measurements yield substantial errors in both the absolute nanobubble concentration and their size distribution.

Therefore, a dilution method using n-hexadecane was employed. A pipette was used to take 10 mL of nanobubble diesel fuel and 10 mL of n-hexadecane liquid. The diesel sample was diluted 1:1 with n-hexadecane, and the diluted sample was measured using the NS300 instrument. As shown in Figure 3b, the system can clearly capture the scattered light spot signal image of nanobubbles in n-hexadecane-diluted diesel. In this image, the scattered light spot signals of individual nanobubbles are distinctly distinguishable. While this method enables clear identification of individual nanobubbles, the dilution process can disrupt the saturated state of gases in the diesel solution. Moreover, some nanobubbles may be damaged during dilution, leading to an underestimation of their actual concentration. Nevertheless, this remains the optimal method currently available for measuring the concentration of nanobubbles in diesel. Sensitivity parameters need to be set on the NS300 instrument, which primarily function to amplify or attenuate the signals captured by the instrument. A lower parameter value may introduce invalid noise, whereas an excessively high value could filter out genuine nanobubbles. For this reason, each sample requires adjustment to an appropriate sensitivity parameter setting. The sensitivity was set within the range of 5 to 7, enabling more accurate measurements. Each experimental sample was measured three times to ensure the accuracy and repeatability of the experimental data. Figure 4b presents the size-resolved concentration distribution of nanobubbles in the system shown in Figure 3b. The results demonstrate that dilution with n-hexadecane significantly improves the measurement accuracy and clarity of the nanobubble size distribution in diesel. This treatment effectively reduces background interference, yielding more reliable and interpretable data. In subsequent experiments, the NS300 instrument will be extensively used to measure the concentration of nanobubbles. The Supplementary Materials include scattered light spot signal image of nanobubbles, which were captured by the NS300 under different experimental conditions.

## 3. Results and Discussion

### 3.1. Blank Experiment

Pure diesel fuel inherently contains nanoparticle impurities and low-concentration nanobubbles. These fuel oils are derived from the hydrorefining and storage tank units of the refinery. As the fuel comes into long-term contact with metal containers, although the specific types and compositions of the nanoparticles present remain unknown, it is reasonable to infer that nanoparticle impurities are present. These particles typically range in size from 100 to 1000 nanometers, with the majority falling between 300 and 600 nanometers. The Malvern Nano Particle Tracking Analysis Instrument NS300 is capable of detecting nanoscale particles but cannot differentiate between nanobubbles and nanoparticles. To ensure that the NS300 instrument measures only nanobubbles, experiments were conducted to verify the presence of nanobubbles.

**Figure 5:**
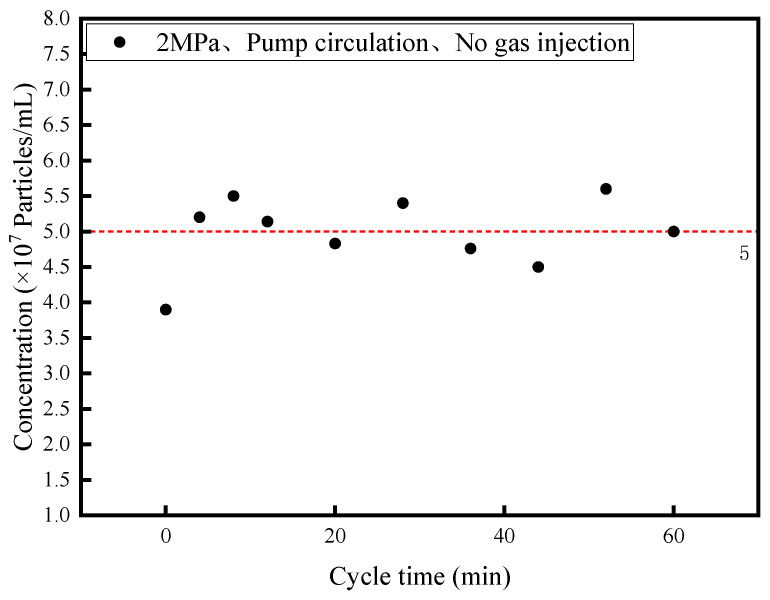
Nanobubble concentration along with cycle time for 2 MPa non-gas injection.

At the beginning of the experiment, nitrogen was introduced into the diesel tank to increase the internal pressure to 2 MPa. Without introducing any additional gases or using the bubbling tube for gas dissolution, the diesel circulation pump was activated, and samples were collected at specific time intervals for concentration measurement. As depicted in Figure 5, the curve illustrates the relationship between nanoparticle concentration and time. Under the condition of no gas bubbling or dissolution, as the diesel circulation time increased, the nanoparticle concentration fluctuated around 5 × 10^7^ particles/mL. This confirms that when no gas is bubbled into the diesel, the inherent nanoparticle concentration in the fuel remains at 5 × 10^7^ particles/mL. The PTA picture under this experimental condition can be found in the Supplementary Materials under “The PTA picture of nanobubble concentration along with cycle time for 2MPa non-gas injection”.

### 3.2. Effect of Cycle Time

**Figure 6:**
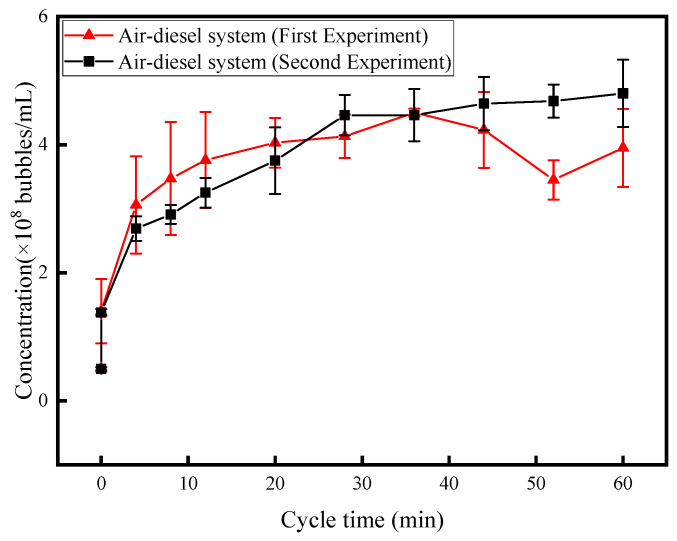
Nanobubble concentration along with cycle time for 2 MPa normal operation.

To measure the variation in nanobubble concentration with circulation time in an air–diesel system, air was introduced into the diesel. During the experiment, the tank internal pressure was maintained at 2 MPa, the diesel liquid flow rate was set to 15 L/min, and the air gas flow rate was adjusted to 50 mL/min. The experiment was repeated twice to ensure accuracy and repeatability. Figure 6 shows the curve illustrating the relationship between circulation time and nanobubble concentration in the diesel fuel.

The time point at *t* = 0 corresponds to the moment when air had been bubbled into the diesel for 30 min but the circulation pump was not activated. At this stage, the air solubility in diesel is assumed to have reached near-saturation. Thirty minutes of air bubbling increased the system pressure, promoting gas dissolution and raising the nanobubble concentration from 5 × 10^7^ ± 4.2 × 10^6^ bubbles/mL to 1.4 × 10^8^ ± 5 × 10^7^ bubbles/mL. Upon activating the diesel pump for cyclic enrichment, the nanobubble concentration grew rapidly during the first 20 min, escalating from 1.4 × 10^8^ ± 5 × 10^7^ bubbles/mL to 4 × 10^8^ ± 3.9 × 10^7^ bubbles/mL. Although the concentration continued to increase in the subsequent 40 min, the growth rate decelerated significantly, rising from 4 × 10^8^ ± 3.9 × 10^7^ bubbles/mL to 4.5 × 10^8^ ± 0.6 × 10^7^ bubbles/mL before stabilizing at this level. While the first experiment showed potential sampling bias, the nanobubble concentration profile in the second experiment exhibited more consistent and plausible trends.

From the results, it is evident that the nanobubble concentration in diesel fuel increases with circulation time, featuring a rapid rise within the first 20 min followed by a decelerated growth in the subsequent 40 min. Notably, there exists a maximum threshold for nanobubble concentration enhancement. Once the nanobubble concentration reaches its threshold, it will no longer increase with prolonged circulation time. In the 2 MPa air–diesel system, the nanobubble concentration can reach up to 4.5 × 10^8^ ± 0.6 × 10^7^ bubbles/mL.

### 3.3. Effect of Pressure

**Figure 7:**
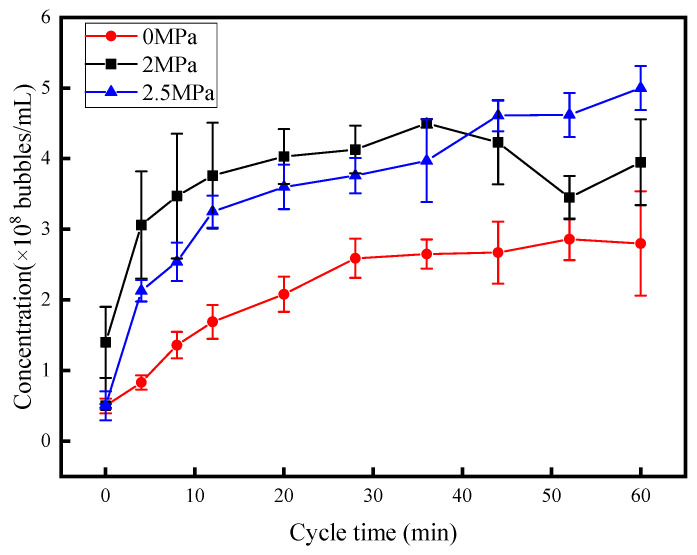
Influence of operation pressure on nanobubble concentration.

Figure 7 illustrates the variation in nanobubble concentration under different ambient pressures in the air–diesel system, comparing the results across various pressure conditions. The maximum nanobubble preparation concentrations are 2.86 × 10^8^ ± 2.9 × 10^7^ bubbles/mL at 0 MPa, 4.5 × 10^8^ ± 0.6 × 10^7^ bubbles/mL at 2 MPa, and 5 × 10^8^ ± 3.1 × 10^7^ bubbles/mL at 2.5 MPa. As the pressure increased from 0 MPa to 2.5 MPa, the maximum achievable concentration of nanobubbles rose from 2.86 × 10^8^ ± 2.9 × 10^7^ bubbles/mL to 5 × 10^8^ ± 3.1 × 10^7^ bubbles/mL, representing a 74.8% increase. These results confirm that elevating the ambient pressure can significantly enhance the concentration of nanobubbles in diesel fuel.

Elevating the experimental pressure significantly enhances the solubility of air in diesel fuel—a key phenomenon rooted in Henry’s law, which dictates that gas solubility in a liquid increases proportionally with applied pressure under constant temperature. As the solubility of air rises, more gas molecules dissolve into the diesel matrix, creating a higher concentration of dissolved gas species. This surplus of dissolved gases amplifies both the probability and quantity of gas-to-nanobubble transitions: with more gas molecules available, the likelihood of nucleation (the initial formation of nanobubble nuclei) increases, while the greater abundance of dissolved gas ensures a larger pool of molecules to feed the growth and stabilization of these nascent nanobubbles. Collectively, these effects drive a substantial increase in the concentration of air nanobubbles dispersed within the diesel fuel. The PTA picture under this experimental condition can be found in the Supplementary Materials under “The PTA picture of the concentration of 0 MPa nanobubbles”, “The PTA picture of the concentration of 2 MPa nanobubbles” and “The PTA picture of the concentration of 2.5 MPa nanobubbles”.

### 3.4. Stability of Nanobubbles

The stability of nanobubbles was evaluated by monitoring the concentration decay of prepared diesel nanobubbles over time. This experiment utilized nanobubble-loaded diesel prepared in a 2 MPa air–diesel system, with a diesel liquid flow rate of 15 L/min and air flow rate of 50 mL/min during synthesis. Samples collected at circulation times of 12 min and 60 min were selected to characterize nanobubble concentration decay over a 2-month period. Concentration measurements were performed daily during the first month and every 4 days in the second month. The stability of nanobubbles was analyzed based on the decay profiles of these two samples over the 2-month observation period.

**Figure 8:**
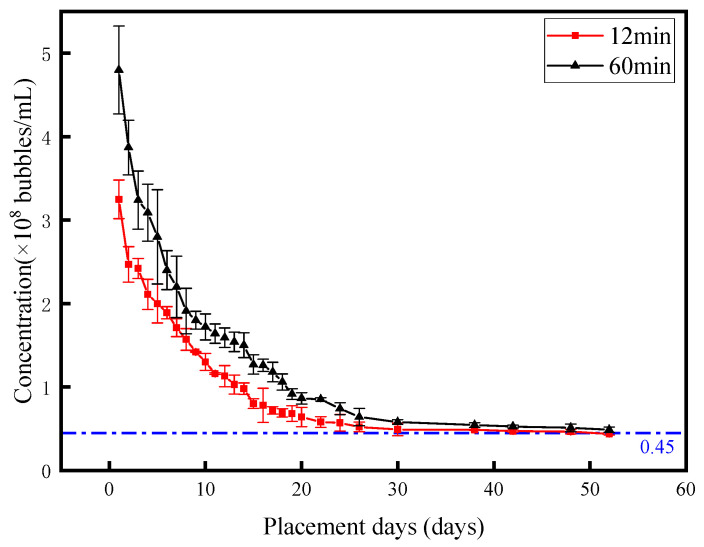
The relationship between the concentration of nanobubbles and the standing time.

As illustrated in Figure 8, the curves depict the two-month decay profiles of nanobubble concentration for the two samples. The decay rate was most pronounced during the first week: the 12 min sample concentration dropped from 3.25 × 10^8^ ± 2.32 × 10^7^ bubbles/mL to 1.71 × 10^8^ ± 1.07 × 10^7^ bubbles/mL, while the 60 min sample decreased from 4.8 × 10^8^ ± 5.26 × 10^7^ bubbles/mL to 2.2 × 10^8^ ± 3.68 × 10^7^ bubbles/mL—both exhibiting approximately 50% reductions. Notably, the concentration order of magnitude remained unchanged, indicating persistent high-concentration nanobubble populations.

After the first week, the decay rate gradually decelerated, with an average daily decline of 8%. By the end of the two-month period, both samples stabilized at a nanoparticle concentration of 4.5 × 10^7^ ± 3.13 × 10^6^ bubbles/mL—comparable to the baseline concentration in pure diesel—confirming minimal residual nanobubble presence. Overall, nanobubble concentration decreases with storage time, characterized by rapid decay in the initial week followed by gradual decline, eventually approaching the 4.5 × 10^7^ particles/mL baseline typical of undoped diesel fuel. The PTA picture under this experimental condition can be found in the Supplementary Materials under “The PTA picture of 12-min sample stability test” and “The PTA picture of 60-min sample stability test”.

### 3.5. Physical Properties of Diesel Fuel Containing Nanobubbles

This experiment explored the influence of nanobubble concentration on key physical properties of diesel fuel, including density, viscosity, and surface tension. The effect of nanobubble concentration on diesel density and viscosity was measured at 18.9 °C and 25 °C, respectively, with its impact on surface tension further analyzed at 18.9 °C.

**Figure 9:**
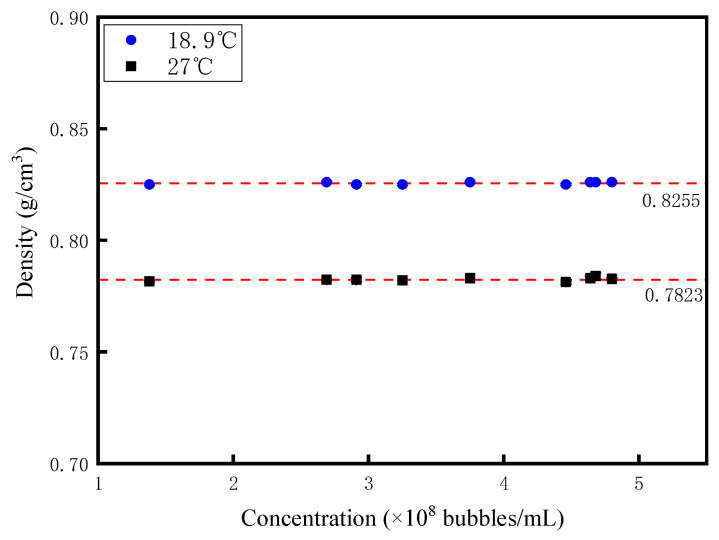
The relationship between the concentration of nanobubbles and diesel density.

The density of nanobubble diesel was measured using a liquid densitometer (model: MDJ-300Y, Xiamen Xiongfa Instrument Co., Ltd., Xiamen, China). Figure 9 illustrates the relationship between nanobubble concentration and diesel density. Under isothermal conditions, no significant variation in diesel density was observed with changes in nanobubble concentration. Specifically, at 18.9 °C, the diesel density measured 0.8255 ± 4.7 × 10^−4^ g/cm^3^, while increasing the temperature to 27 °C caused the density to decrease to 0.7823 ± 9.4 × 10^−4^ g/cm^3^. These results indicate that the influence of nanobubble concentration on diesel density is negligible, whereas temperature elevation significantly reduces diesel density—consistent with the general principle of fluid thermal expansion. It is worth noting that the error bars for the experimental data regarding the density of nanobubble diesel have been marked in Figure 9. However, owing to the small standard deviation of the experimental data, these error bars are extremely short and thus cannot be clearly observed in the figure.

**Figure 10:**
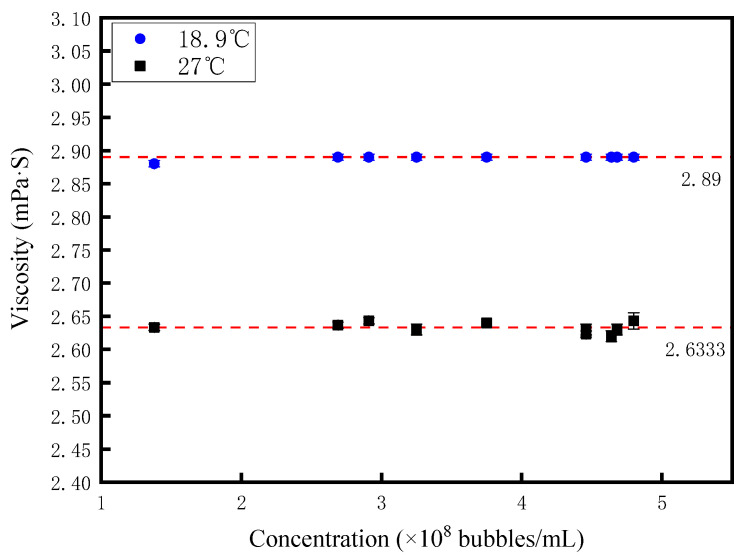
The relationship between the concentration of nanobubbles and diesel viscosity.

The viscosity of nanobubble diesel was determined using a rotational viscometer (model: NDJ-1S, Shanghai Hengping Instrument and Meter Factory, Shanghai, China). Figure 10 depicts the correlation between nanobubble concentration and diesel viscosity. At constant temperature, no significant viscosity variation was observed with changes in nanobubble concentration. Specifically, at 18.9 °C, the viscosity measured 2.890 ± 4.7 × 10^−3^ mPa·s, decreasing to 2.633 ± 8.16 × 10^−3^ mPa·s at 27 °C (an ~8.9% reduction). Analogous to density, temperature emerges as the primary determinant of viscosity: increasing temperature diminishes intermolecular forces, thereby reducing viscosity, while the influence of nanobubble concentration remains negligible. Similarly, the error bars for the viscosity experimental data of nanobubble diesel are also marked in Figure 10. However, due to the small standard deviation of the experimental data, these error bars are extremely short and therefore cannot be clearly observed in the figure.

**Figure 11:**
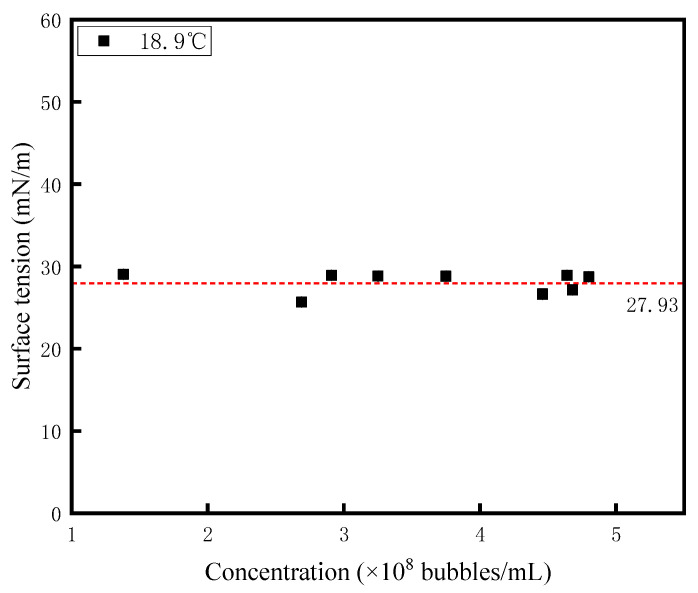
The relationship between the concentration of nanobubbles and diesel surface tension.

The surface tension of nanobubble diesel is measured using a fully automatic surface tension meter (model: YP-ZL1, Shanghai Yupeng Electronic Technology Co., Ltd., Shanghai, China). Figure 11 illustrates the relationship between nanobubble concentration and diesel surface tension, revealing that nanobubble concentration exerts a relatively limited effect on this property. While minor fluctuations in surface tension were observed at different concentrations, the values remained stable within the range of 27.93 ± 0.065 mN/m, demonstrating no significant concentration-dependent behavior. Though the concentration of the prepared nanobubbles was relatively high, with levels reaching 10^8^ bubbles/mL, their volume proportion relative to the total volume of the diesel fuel remained extremely small. As a result, they were limited in their ability to significantly affect the fuel’s surface tension, and the overall influence was quite negligible. Similarly, the error bars for the surface tension experimental data of nanobubble diesel are also marked in Figure 11. However, due to the small standard deviation of the experimental data, these error bars are extremely short and therefore cannot be clearly observed in the figure.

## 4. Conclusions

To tackle the challenge of measuring nanobubble concentration in opaque diesel fuel, this study adopted a dilution approach using n-hexadecane at a 1:1 ratio, combined with quantification via the Malvern NS300 Nanoparticle Tracking Analyzer. While this testing method may not represent an absolutely precise means of verifying nanobubble concentration, it currently stands as a reasonable and feasible solution for measuring nanobubble concentration in diesel.

The experimental results demonstrated that the nanoparticle concentration in diesel prepared without gas bubbling matched that of pure diesel, whereas gas bubbling dissolution followed by circulation significantly enhanced nanoparticle concentration—validating the system’s efficacy in generating high-concentration nanobubble-laden diesel. To optimize nanobubble generation, the impacts of circulation time and pressure were systematically examined. The results indicated that nanobubble concentration increased with circulation time until a saturation threshold was reached. Elevated pressure enhanced gas solubility and nanobubble conversion efficiency, yielding higher concentrations: specifically, under 2 MPa and 60 min of circulation, nanobubble concentration peaked at 4.8 × 10^8^ ± 5.26 × 10^7^ bubbles/mL.

Stability analysis over a two-month period revealed a biphasic decay pattern: an initial rapid decline (50% reduction within one week) followed by a slower deceleration (8% daily decrease), eventually stabilizing at the background concentration of pure diesel (4.5 × 10^7^ ± 3.13 × 10^6^ bubbles/mL). These findings highlight that while nanobubbles exhibit transient stability, optimal conditions sustain high concentrations (>10^8^ bubbles/mL) over extended durations, underscoring the practical feasibility of this generation method for applications requiring persistent nanobubble functionality in diesel fuel systems.

Further investigations into the physical properties of diesel revealed negligible effects on density and viscosity across all nanobubble concentrations. However, surface tension exhibited a minor inverse correlation with nanobubble concentration, decreasing by up to 3.2% at peak concentrations. Although the concentration of the prepared nanobubbles is relatively high, reaching 10^8^ bubbles/mL, their volume and mass ratio relative to the total volume and mass of diesel remain extremely small. It can thus be inferred that the incorporation of nanobubbles into diesel has little to no impact on its physical properties and consequently exerts a negligible or even non-existent effect on the fuel’s density, viscosity, and surface tension.

## 5. Patents

Chinese invention patent with a public status: CN202411959563.3 A method and device for preparing high-concentration nanobubble fuel.

## Data Availability

The original contributions presented in this study are included in the article/Supplementary Materials. Further inquiries can be directed to the corresponding author.

## Supplementary Materials

The following supporting information can be downloaded at https://www.mdpi.com/article/10.3390/nano15171309/s1. The PTA picture.

## Abbreviations

The following abbreviations are used in this manuscript:

CO_2_	carbon dioxide
NO_x_	nitrogen oxides
SO_x_	sulfur oxides
PM	particulate matter
HC	unburned hydrocarbons
BSFC	brake-specific fuel consumption
HNB	hydroxygen nanobubble
rpm	revolutions per minute
