# Supplementary material for: Preparation of Air Nanobubble-Laden Diesel
Source: Nanomaterials (Basel). 2025 Aug 25;15(17):1309. doi: 10.3390/nano15171309 (PMC12430541; doi:10.3390/nano15171309)
Supplement: Supplementary file 1 [file nanomaterials-15-01309-s001.zip › nanomaterials-3748917-SI.pdf]

Supplementary Materials

The PTA picture of nanobubble concentration along with cycle time for 2MPa non-gas injection

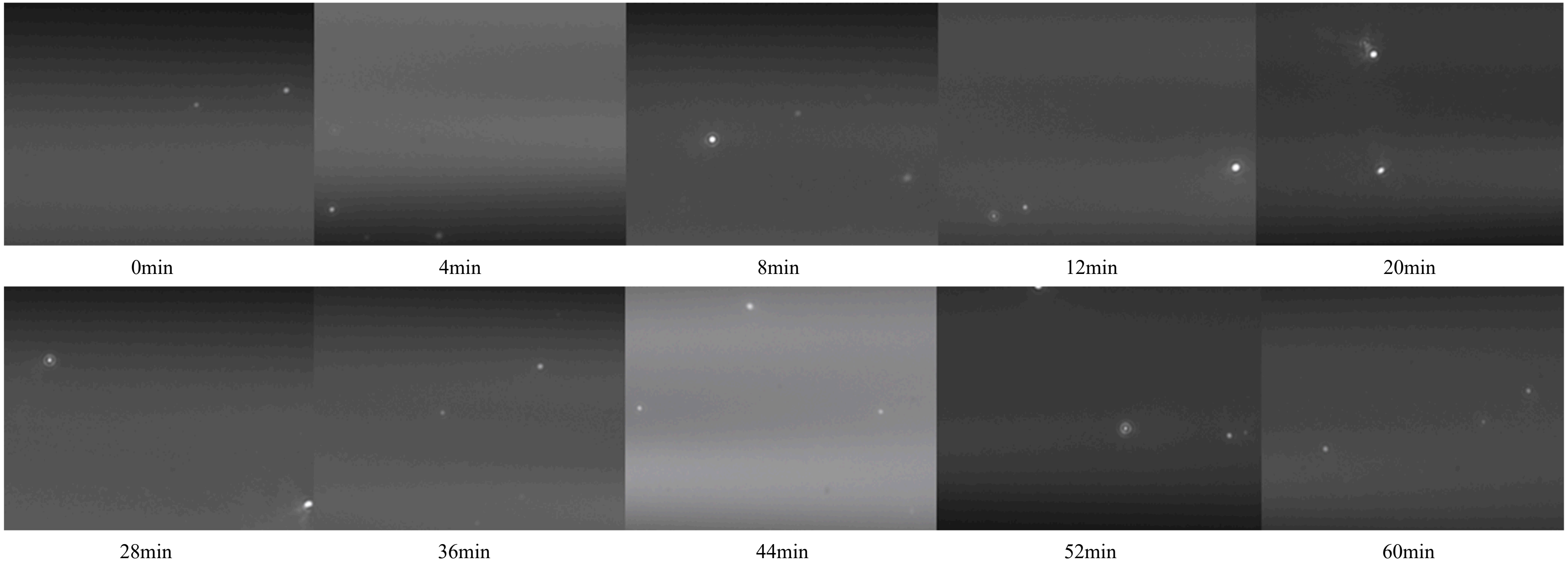

The PTA picture of the concentration of 0 MPa nanobubbles

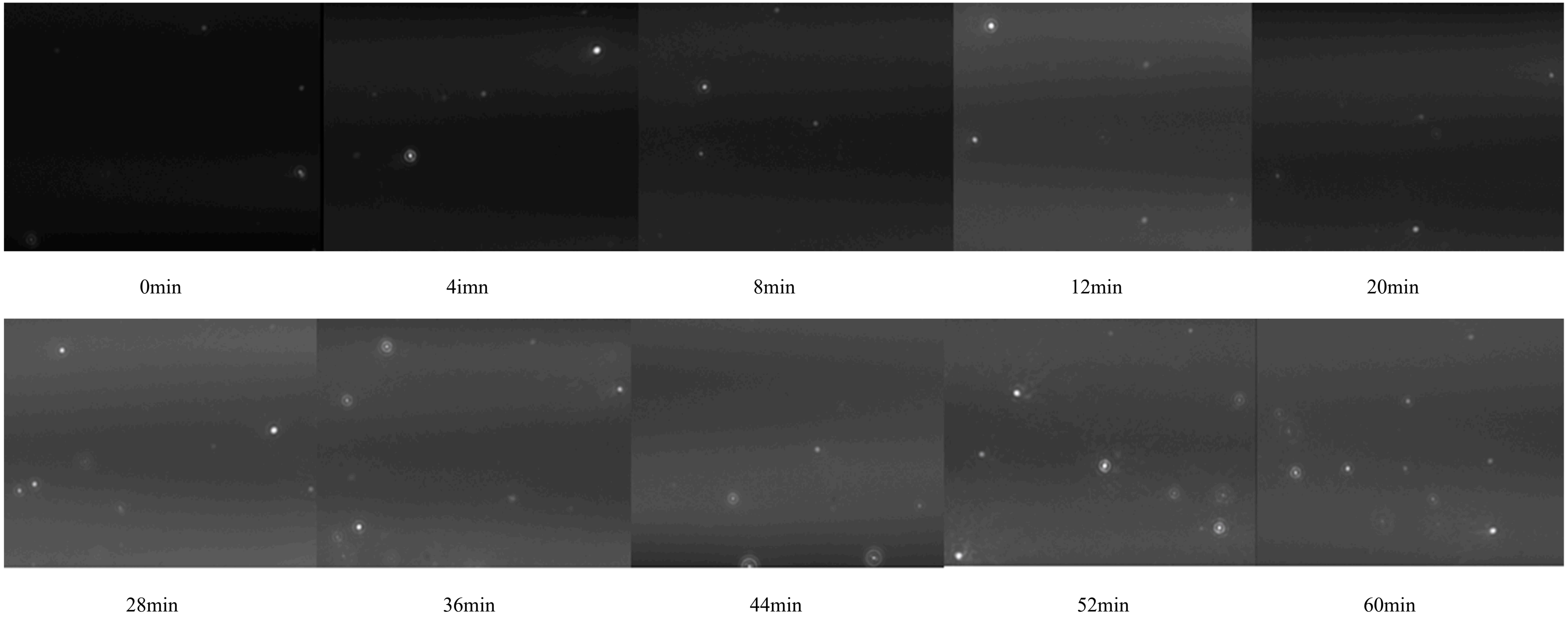

The PTA picture of the concentration of 2 MPa nanobubbles

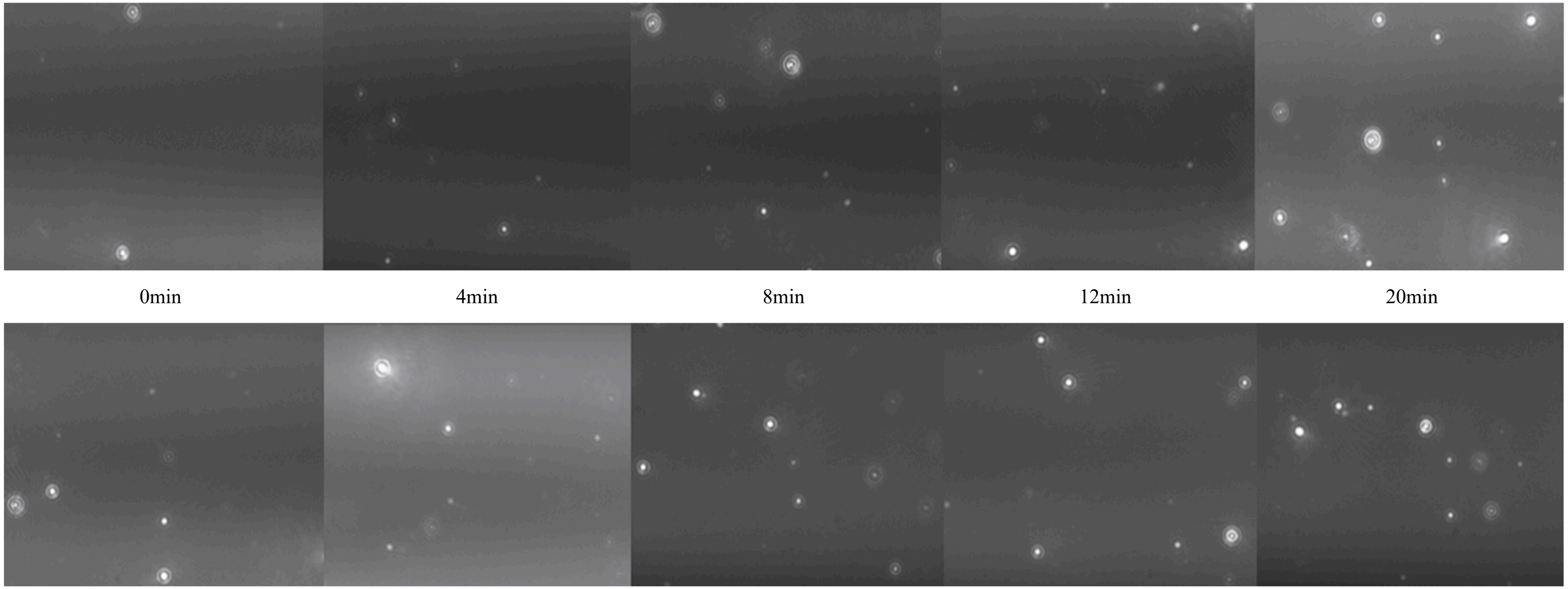

The PTA picture of the concentration of 2.5 MPa nanobubbles

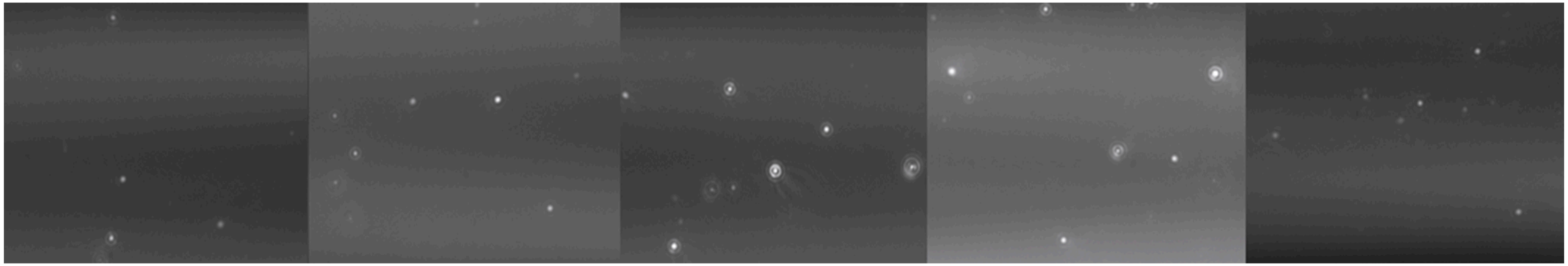

0min 4min 8min 12min 20min

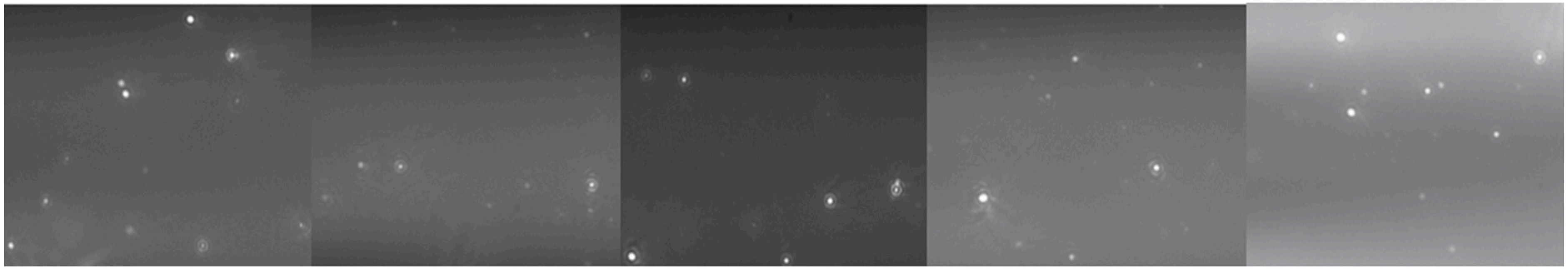

28min 36min 44min 52min 60min

The PTA picture of 12-minute sample stability test

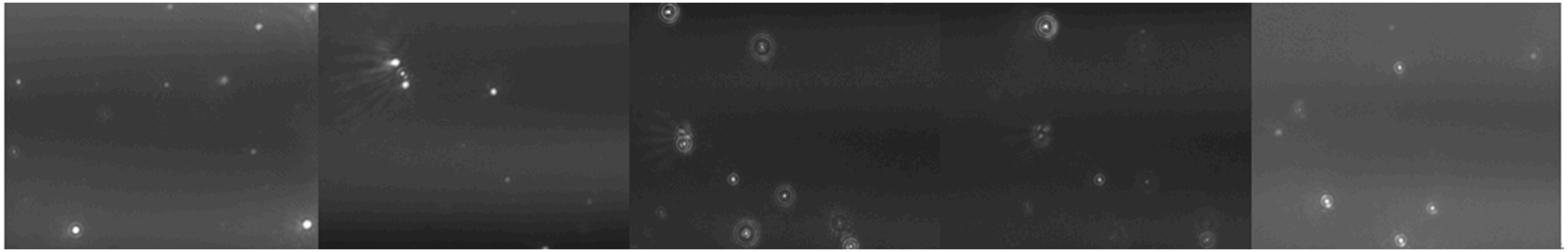

DAY 1 DAY 2 DAY 3 DAY 4 DAY 5

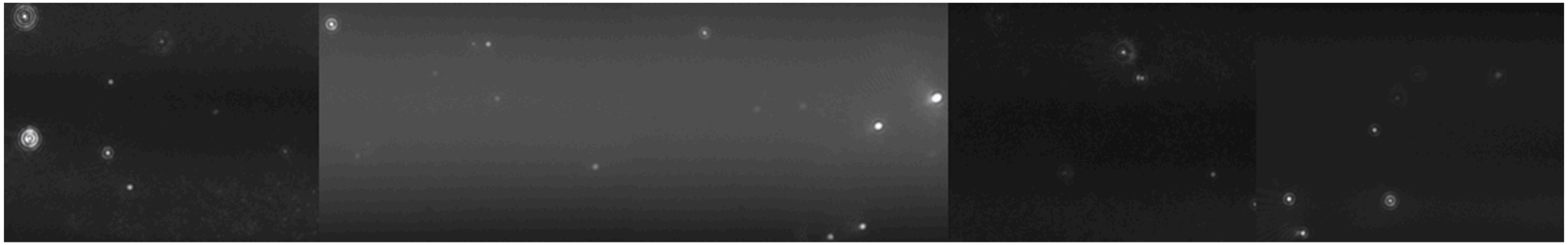

DAY 6 DAY 7 DAY 8 DAY 9 DAY 10

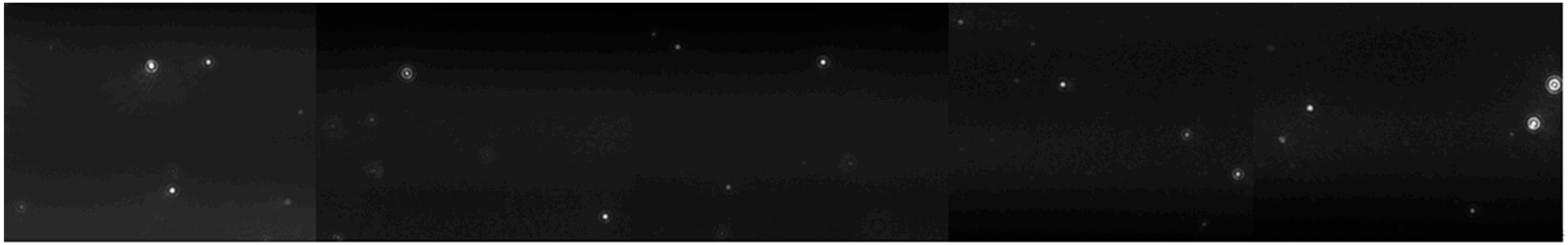

DAY 11 DAY 12 DAY 13 DAY 14 DAY 15

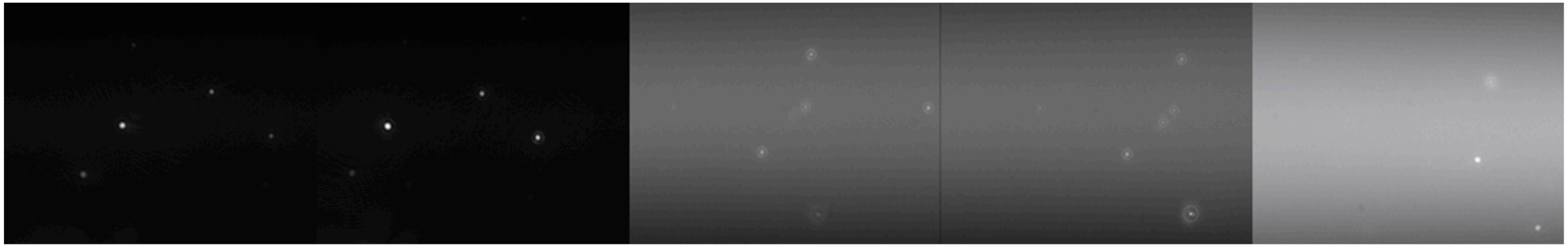

DAY 16 DAY 17 DAY 18 DAY 19 DAY 20

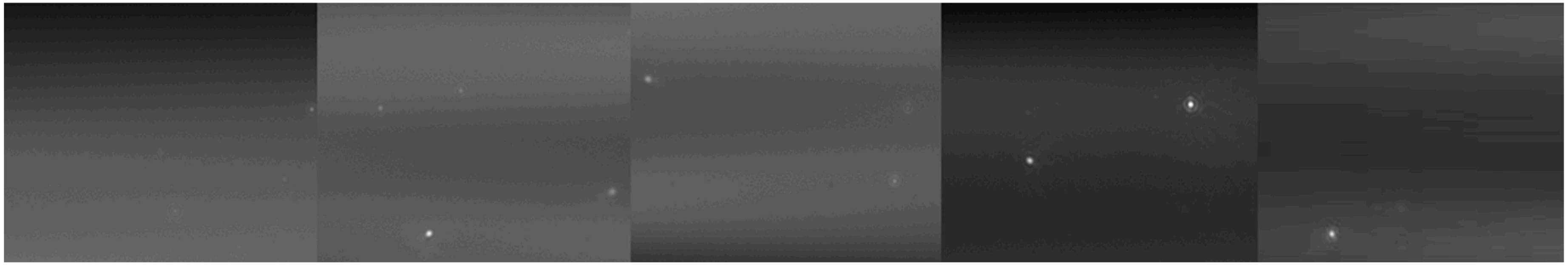

DAY 22 DAY 24 DAY 26 DAY 30 DAY 38

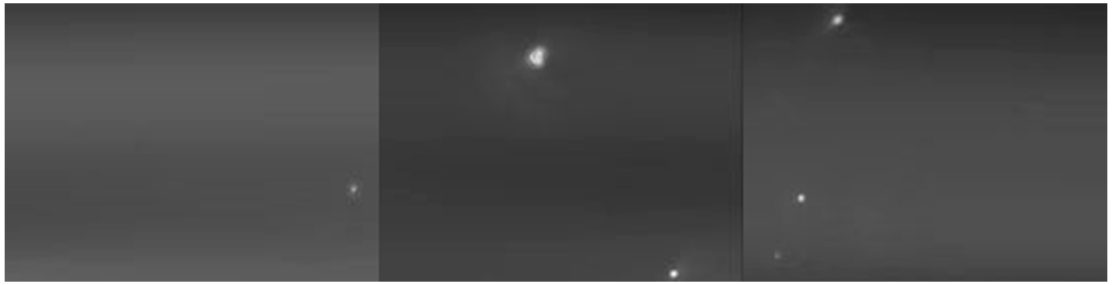

DAY 42 DAY 48 DAY 52

The PTA picture of 60-minute sample stability test

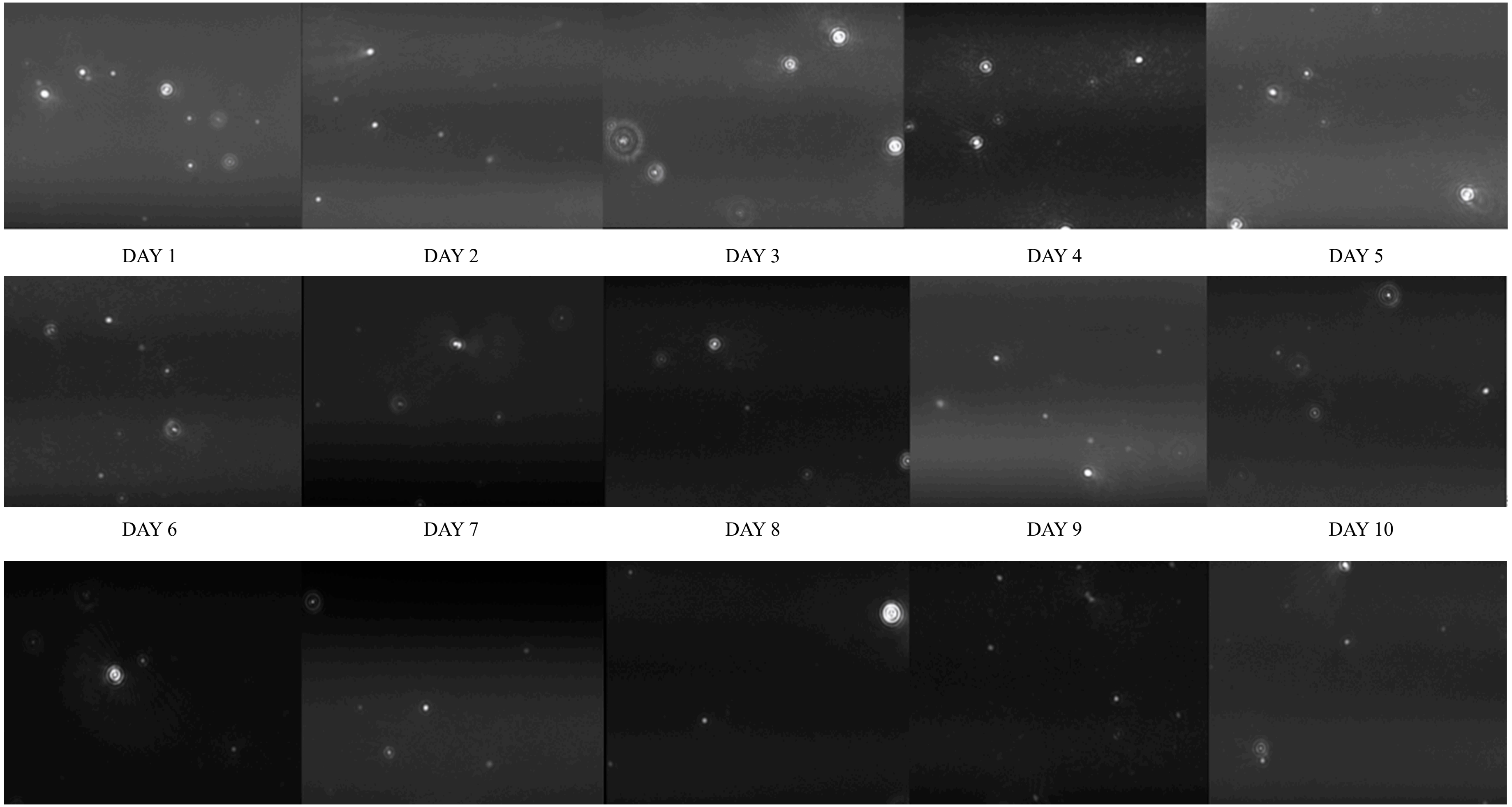

DAY 1 DAY 2 DAY 3 DAY 4 DAY 5

DAY 6 DAY 7 DAY 8 DAY 9 DAY 10

DAY 11 DAY 12 DAY 13 DAY 14 DAY 15

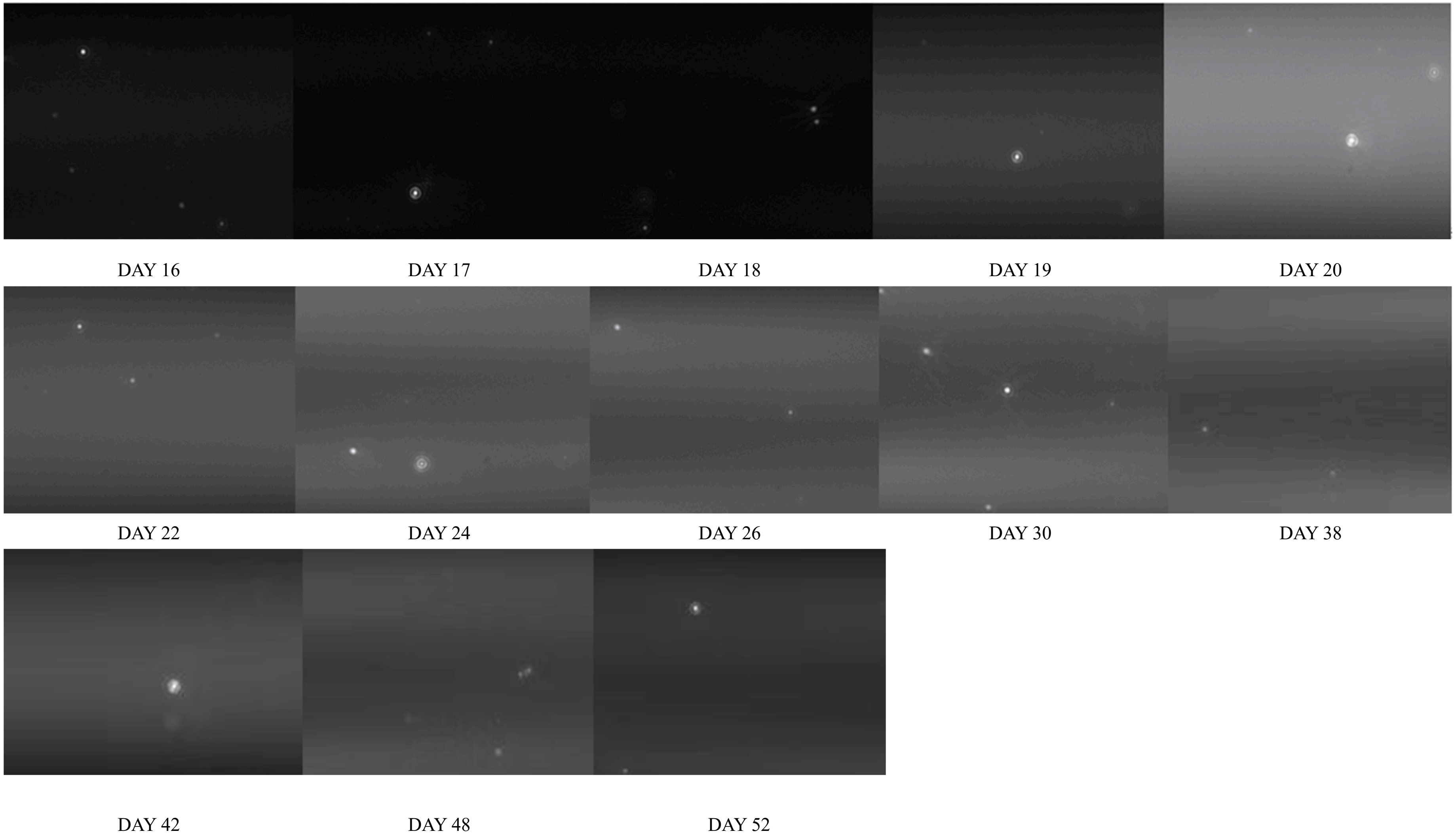
DAY 16
DAY 17
DAY 18
DAY 19
DAY 20
DAY 22
DAY 24
DAY 26
DAY 30
DAY 38
DAY 42
DAY 48
DAY 52
